# 9-*cis*-epoxycarotenoid dioxygenase 1 confers heat stress tolerance in rice seedling plants

**DOI:** 10.3389/fpls.2022.1092630

**Published:** 2022-12-20

**Authors:** Yijin Zhang, Xiong Liu, Rui Su, Yunhua Xiao, Huabing Deng, Xuedan Lu, Feng Wang, Guihua Chen, Wenbang Tang, Guilian Zhang

**Affiliations:** ^1^ College of Agronomy, Hunan Agricultural University, Changsha, China; ^2^ Hunan Provincial Key Laboratory of Rice and Rapeseed Breeding for Disease Resistance, Changsha, China; ^3^ Hunan Hybrid Rice Research Center, Hunan Academy of Agricultural Sciences, Changsha, China; ^4^ State Key Laboratory of Hybrid Rice, Changsha, China

**Keywords:** rice, seedling, heat stress, ABA, *OsNCED1*

## Abstract

High temperature is one of the main constraints affecting plant growth and development. It has been reported that abscisic acid (ABA) synthesis gene *9-cis-epoxycarotenoid dioxygenase* (*NCED*) positively regulates plant resistance to salt, cold, and drought stresses. However, little is known about the function of the *NCED* gene in heat tolerance of rice. Here, we found that *OsNCED1* was a heat stress inducible gene. Rice seedlings overexpressing *OsNCED1* showed enhanced heat tolerance with more abundant ABA content, whereas the knockout mutant *osnced1* accumulated less ABA and showed more sensitive to heat stress. Under heat stress, increased expression of *OsNCED1* could reduce membrane damage and reactive oxygen species (ROS) level of plants, and elevate the activity of antioxidant enzymes. Moreover, real time-quantitative PCR (RT-qPCR) analysis showed that overexpression of *OsNCED1* significantly activated the expression of genes involved in antioxidant enzymes, ABA signaling pathway, heat response, and defense. Together, our results indicate that *OsNCED1* positively regulates heat tolerance of rice seedling by raising endogenous ABA contents, which leads to the improved antioxidant capacity and activated expression of heat and ABA related genes.

## Introduction

Rice (*Oryza sativa* L.) is a major food crop cultivated in countries around the world, especially in Asia, and feeds more than 50% of the global population. Rising temperature due to global warming has a serious impact on rice production, and the damage may continue to rise in the future, particularly for Asian rice (Qin-Di et al., 2021). Growing rice above its optimal growth temperature of 5°C results in a corresponding thermal response profile at the cellular and metabolic levels to maintain its own survival and growth (Barnabás et al., 2008). Heat stress is an essential environmental factor limiting rice growth and reproduction, causing different damage to different development stages of rice, and it will suffer from different degrees of heat stress from germination to seedling, anthesis, grain filling, and grain maturation stages (Qin-Di et al., 2021). It has been reported that rice yield decreases by 3.2% for every 1°C increasing in global temperature (Zhao et al., 2017). The difficulty of rice to maintain its normal ontogeny under high temperature conditions, including photosynthesis, respiration, enzyme activity, formation of organs of both sexes, nutrient uptake, and so on, which is the main reason why it affects rice yield (Barnabás et al., 2008).

Abscisic acid (ABA) is a sesquiterpenoid with 15 carbon atoms, which is synthesized indirectly by the carotenoid pathway in plants. The synthetic path of ABA is that zeaxanthin epoxidase (ZEP) catalyzes zeaxanthin (C_40_) to all-trans-violaxanthin, then the neoxanthin synthase (NSY) converts all-trans-violaxanthin to 9’-*cis*-neoxanthin, and finally the *cis* isomer is cracked by 9-*cis*-epoxycarotenoid dioxygenase (NCED) and product C_15_ xanthotoxin, which is an important step of ABA biosynthesis (Chen et al., 2020). ABA plays a crucial role in regulating plant stress responses. Its biosynthesis is induced by several environmental stresses, such as drought, salt, and cold stress (Nambara and Marion-Poll, 2005). Studies have shown that the bloating germination of *Arabidopsis thaliana* seeds at elevated temperatures is associated with the induction of elevated ABA levels by the zeaxanthin epoxidase gene *ABA1* and the three 9-*cis*-epoxycarotenoid dioxygenase genes *NCED2*, *NCED5* and *NCED9* (Toh et al., 2008). Heat stress decreases auxin and gibberellin content, and increases endogenous ABA content in rice anthers (Tang et al., 2007). Exogenous ABA maintains carbon and energy balance of rice by increasing sucrose transport and accelerating sucrose metabolism, to prevent pollen abortion under heat stress, and improve seed setting rate (Rezaul et al., 2019). The bZIP transcription factor gene of the ABA signaling pathway, *ZmbZIP4*, can regulate ABA synthesis related genes to promote the synthesis of ABA to improve high temperature tolerance in maize (Zea mays) seedlings (Ma et al., 2018). The above findings indicate that ABA is involved in plant response to high-temperature stress, but the underlying mechanism of the effect of heat stress on endogenous ABA content in rice is still unclear.

9-*cis*-epoxycarotenoid dioxygenase (NCED) is a key rate limiting enzyme in ABA biosynthesis, and its activity affects endogenous ABA accumulation in plants (Kalladan et al., 2019). Overexpression of *NCED* promotes ABA synthesis in response to various abiotic stresses (Zhang et al., 2014; Huang et al., 2018), whereas knockdown of *NCED* reduces ABA accumulation, and shows abiotic stress sensitive (Frey et al., 2012). The first *NCED* gene to be discovered was *viviparous 14* (*VP14*) in a maize mutant (Nambara and Marion-Poll, 2005), and *NCED* genes were subsequently isolated from other plant species, such as tomato (*Solanum lycopersicum*), *Arabidopsis*, and apple (Huang et al., 2018).

There are five members of the *NCED* family in rice, *OsNCED1*, *OsNCED2*, *OsNCED3*, *OsNCED4* and *OsNCED5* (Zhu et al., 2009). *OsNCED1* expression is elevated by salt and drought stress but repressed by water stress (Huang et al., 2018; Jiang et al., 2019). It has also been shown that the cis-acting element ABRE region exists in the *AhNCED1* promoter, and ABA treatment under water stress can increase promoter activity, promote *AhNCED1* gene and protein expression, and promote ABA synthesis to enhance water stress (Liang et al., 2009), and that the ectopic expression of *AhNCED1* will improve ABA accumulation to improve water stress tolerance in *Arabidopsis* plants (Wan and Li, 2006). In addition, *OsNCED1* could indirectly regulate the seed setting rate of rice under low temperature stress at flowering stage by regulating ABA metabolic pathway (Huang et al., 2018). Both *OsNCED2* and *OsNCED3* expression levels correlate with delayed seed germination (Zhu et al., 2009; Song et al., 2012). *OsNCED3* regulates seed dormancy, stomatal opening, plant growth and leaf senescence by altering ABA accumulation in rice (Huang et al., 2018). In addition, *OsNCED3*, *OsNCED4* and *OsNCED5* induce the expression and promote ABA production under water stress, which would affect plant growth and water stress (Teng et al., 2014; Zhang et al., 2015). We have reported that overexpression of *OsNCED1* improved high temperature stress tolerance at heading and anthesis stages of rice (Zhou et al., 2022). However, the roles of *OsNCED1* under heat stress in rice seedling stage remains unclear.

At present, little is known about the function of genes involved in the ABA synthesis pathway and signal regulation pathway in heat stress responses in rice. In this study, we constructed *OsNCED1* overexpressing and gene edited transgenic rice plants, and aimed to explore the biological function of *OsNCED1* under heat stress at the seedling stage of rice.

## Materials and methods

### Plant materials

The rice japonica variety Nipponbare (Nip), *OsNCED1* overexpression lines (Nip), 252 and *osnced1* mutants (252) were used in this study. 252 was an extreme heat tolerant individual of the recombinant inbred lines derived from a cross between the heat-tolerant rice line 996 and the sensitive line 4628. The overexpression vectors (pCAMBIA1300-*OsNCED1*) and gene editing vectors (pBWA(V)HU-ylcas9-*osnced1*) were constructed, and respectively transformed into *Agrobacterium tumefaciens* EHA105, and then introduced into Nip and 252 plants. Positive transgenic lines were selected and further identified by PCR and real time-quantitative PCR. Overexpression lines (*OE-1* and *OE-2*) with higher transcript levels were used for further analysis, and *osnced1* mutants (*ko-1* and *ko-2*) were also obtained for further analysis by sequencing.

### High temperature treatment

Seeds of wild-type (Nip and 252), *OsNCED1* overexpression lines (*OE-1* and *OE-2*), and *osnced1* mutants (*ko-1* and *ko-2*) were soaked in water at 37°C for 2 d, and sown in a rice seedling box in light incubator, with the temperature was 25°C, the light intensity was 30000 lux, the light cycle was 12/12 h (light/dark), and the relative humidity was 70%, and germinated for 8 d. Subsequently, 8-day-old transgenic lines and wild-type (WT) were transferred to a light incubator at 45°C for 48 h, and moved to a light incubator with 25°C for recovery. Corresponding WT and transgenic lines were grown in a 25°C light incubator and were set as controls. After 7 days of recovery, the phenotypes of the treated and control plants were photographed and the survival rate of seedling was counted. Four biological replicates were performed for each treatment, and 30 seedlings were replicated for each treatment.

### Measurement of abscisic acid content

Eight-day-old transgenic lines and WT rice plants were transferred to a light incubator at 45°C for 48 h, while the corresponding WT and transgenic seedlings were placed in a light incubator with 25°C as controls. The corresponding WT and transgenic plants were sampled for the determination of ABA content before and 48 h after treatment, and the determination method was referred to the method as described previously (Huang et al., 2018). Briefly, 0.1 g of fresh plants was extracted with 1.5 ml of phytohormone extraction buffer and 2 ng of ABA-d6 internal standard was added, then sample was freeze-dried in nitrogen for 2 times. The ABA levels were quantified by liquid chromatography-tandem mass spectrometry (LC-MS/MS).

### Measurement of physiological indexes

Eight-day-old transgenic lines and WT rice seedlings were treated in a light incubator and exposed to heat stress (45°C) for 48 h, and those in a light incubator at 25°C served as controls. After 48 h of treatment, plants under high temperature stress and control conditions were used for the determination of relative electrolyte leakage rate, malondialdehyde (MDA) content, hydrogen peroxide (H_2_O_2_) content and superoxide anion (O_2_^-^) content, superoxide dismutase (SOD) activity, peroxidase (POD) activity, and three biological replicates were performed. The electrolyte leakage rate conductivity of heat-treated and control plants was measured by the conductivity meter (DS-11A), the MDA content was measured by the thiobarbituric acid (TBA) colorimetric method, the SOD activity was measured by the riboflavin nitro blue tetrazolium (NBT) method, and the POD activity was measured by the guaiacol method. The electrolyte leakage rate, MDA content and antioxidant enzyme activities were determined by the method of Zhou et al. (2022), with a slight modification. Referring to the method of Sun et al. (2019), 3,3-diaminobenzidine (DAB) and nitro blue tetrazolium were used to detect the accumulation of H_2_O_2_ and O_2_^-^ in the leaves of plants. The content of H_2_O_2_ and O_2_^-^ of high temperature treated and control plants were determined using kits (BC3595, Solarbio).

### Quantitative real-time PCR analysis

The WT plants treated with high-temperature for 0 h, 2 h, 4 h, and 8 h, the transgenic and WT plants of high-temperature treated for 48 h and the control were collected, and the corresponding RNA was extracted after snap freezing with liquid nitrogen. RT-qPCR was used to determine the expression levels of *OsNCED1* along with the transcript levels of genes involved in antioxidant enzymes, ABA signaling pathway, heat response, and defense. Total RNA extraction was performed using RNA easy isolation reagent (R701-01, Vazyme) and was reverse transcribed for qPCR analysis using the HiScript^®^ II Q RT SuperMIX for RT-qPCR (+gDNA wiper) kit (R223-01, Vazyme). Rice *OsActin1* was used as an internal reference gene, and primers for amplification were designed by Primer Premier 6.0. The relative changes in gene expression levels were quantitated based on three biological replicates *via* the 2^-ΔΔCt^ method.

### Statistical analysis

All experiments were conducted in three biological replicates. Data are presented as mean ± SE, and statistical analysis was performed using DPS (version 7.05). Data were analyzed by one-way ANOVA and it was considered statistically significant at *p* < 0.05, *p* < 0.01. Plotted using GraphPad Prism (version 8.01).

## Results

### Response of *OsNCED1* to heat stress at rice seedling stage

**Figure 1:**
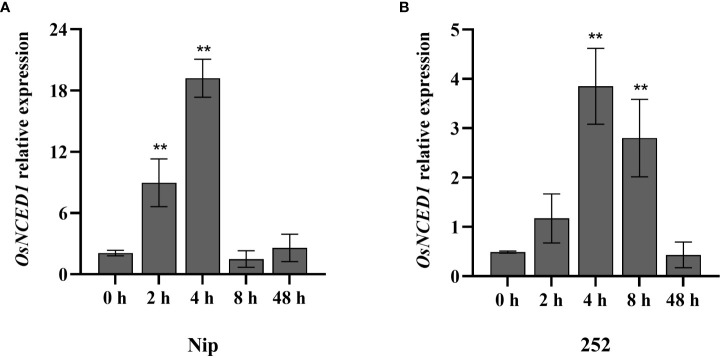
Transcript level of *OsNCED1* in Nip and 252 under heat stress. **(A)** Transcript level of *OsNCED1* in Nip under heat stress. **(B)** Transcript level of *OsNCED1* in 252 under heat stress. Data are means ± SD (n = 3; **P< 0.01, Student’s *t*-test).

In our previous study, the quantitative proteomics technology of iTRAQ quantitative marker combined with LC-MS/MS analysis was used to compare and analyze the difference of anther protein expression between heat tolerant rice line 996 and heat sensitive rice line 4628 under heat stress, and it was found that *OsNCED1* was significantly upregulated (Zhou et al., 2022). In order to further explore the response of *OsNCED1* to heat stress at rice seedling stage, RT-qPCR was used to detect the expression level of *OsNCED1* in seedling of Nip and 252, an extreme heat tolerant individual, at 45°C for 0 h, 2 h, 4 h, 8 h and 48 h. Upon heat stress, the expression of *OsNCED1* increased significantly in Nip and 252, peaked at 4 h and then decreased at 8 h of the heat stress (**Figures 1A, B**). These results indicated that *OsNCED1* responded to heat stress and its expression was strongly induced.

### Effects of heat stress on survival rate and endogenous ABA content of *OsNCED1* transgenic and WT seedlings

**Figure 2:**
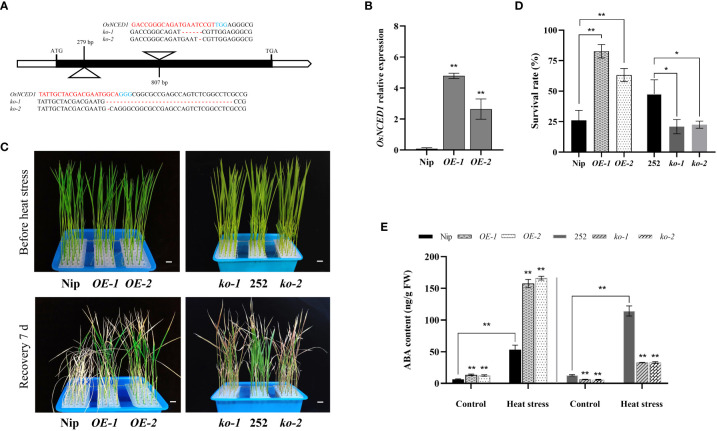
Phenotypic analyses of *OsNCED1* transgenic lines under heat stress. **(A)** The diagram of the two CRISPR/Cas9 target sites, and nucleotide mutation sequences of *ko-1* and *ko-2* lines. The base pairs in blue indicated protospacer adjacent motif (PAM), and the red represented small guide RNA (sgRNA) sequence. **(B)** Real-time quantitative PCR analysis of *OsNCED1* in *OsNCED1* overexpression lines *OE-1* and *OE-2*. Data are means ± SD (n = 3; **P< 0.01, Student’s *t*-test). **(C)** Phenotypes of two WT and *OsNCED1* transgenic rice seedlings before heat stress and 7 days after moderate temperature recovery. Bars = 1 cm. **(D)** Survival rates (%) of two WT and *OsNCED1* transgenic lines after heat stress for 48 h. Data are means ± SD (n = 4; *P< 0.05, **P< 0.01, Student’s *t*-test). **(E)** Endogenous ABA content in WT and *OsNCED1* transgenic lines under control and heat stress. Data are means ± SD (n = 3; **P< 0.01, Student’s *t*-test).

In order to study the role of *OsNCED1* in heat stress, two mutants, *ko-1* and *ko-2*, were constructed in the background of 252 (**Figure 2A**; **Supplementary Figure 1**). The *ko-1* and *ko-2* had two different mutation sites in the *OsNCED1* exons, which caused premature termination of OsNCED1 protein. The overexpression lines *OE-1* and *OE-2* of *OsNCED1* were constructed in the background of Nip. And the expression levels of the overexpression lines *OE*-1 and *OE*-2 were 50.9 times and 30.9 times higher than that of Nip, respectively (**Figure 2B**). As shown in **Figures 2C, D**, upon heat stress, the survival rate of Nip was significantly lower than that of the two overexpression lines (*OE-1* and *OE-2*), while the survival rate of 252 was significantly higher than that of the two mutants (*ko-1* and *ko-2*). Under the control conditions, there is no significant difference between transgenic plants and corresponding WT (**Figure 2D**). The results showed that *OsNCED1* positively regulated the heat stress of rice seedlings.

ABA is in a dynamic equilibrium in response to physiological changes and stimuli from the external environment in plants. NCED is a key rate limiting enzyme for ABA biosynthesis, and affects the accumulation of endogenous ABA (Kalladan et al., 2019). The ABA content under control and heat stress revealed that the ABA levels of the overexpression lines *OE-1* and *OE-2* under control conditions were 13.5 ng/g and 12.4 ng/g, respectively, and significantly higher than that of Nip (6.4 ng/g), whereas the ABA levels of the mutants *ko-1* and *ko-2* were 6.2 ng/g and 5.9 ng/g, respectively, and significantly lower than that of 252 (12.9 ng/g) (**Figure 2E**). The ABA contents of the transgenic and WT plants were significantly increased under heat stress, indicating that heat tolerance of rice might be improved by increasing the ABA content. Thus, *OsNCED1* might improve the tolerance to heat stress by affecting the accumulation of endogenous ABA in rice.

### Changes in membrane lipid peroxidation and antioxidant enzyme activities in *OsNCED1* transgenic and WT seedlings under heat stress

**Figure 3:**
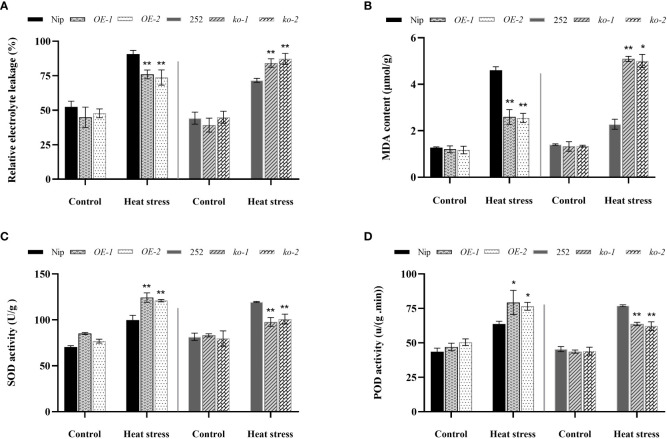
Changes in membrane lipid peroxidation and antioxidant enzyme activities in the transgenic lines and WT plants under heat stress. The electrolyte leakage rate **(A)** and MDA content **(B)** and SOD activity **(C)** and POD activity **(D)** in transgenic lines and WT after 48 h heat stress. Data are means ± SD (n = 3; *P< 0.05, **P< 0.01, Student’s *t*-test).

To reveal the underlying physiological mechanism which *OsNCED1* improves heat tolerance in rice, MDA content, relative electrolyte leakage rate, SOD activity and POD activity of *OsNCED1* transgenic and WT seedling were measured under heat stress. Malondialdehyde can reflect the rate and intensity of peroxidation of plant membrane lipids, and can also indirectly reflect the potential antioxidant capacity of plant cells (Rezaul et al., 2019). Relative electrolyte leakage rate conductivity is an important parameter to examine the membrane permeability of plant cells under abiotic stress (Huang et al., 2018). Under control conditions, there was no significant difference in MDA content and relative electrolyte leakage rate between transgenic and WT seedlings (**Figures 3A, B**). After 48 h of heat stress, the MDA content in both transgenic and WT seedlings increased significantly, but the increase of MDA content and relative electrolyte leakage rate of overexpression lines were significantly lower than that of Nip (**Figures 3A, B**), whereas the increase in the MDA content and relative electrolyte leakage rate of the mutants was significantly higher than that of 252, indicating that the increase in *OsNCED1* can reduce membrane damage in plants under heat stress.

Next, the activities of SOD and POD of transgenic and WT seedlings under heat stress were measured. As shown in **Figures 3C, D**, after 48 h of heat stress, the WT and transgenic lines showed higher activities of both antioxidative enzymes than that of the control, and the increases in antioxidative enzyme activities were significantly greater in the *OE-1* and *OE-2* lines than that of Nip, and the activities of antioxidant enzymes in *ko-1* and *ko-2* lines were significantly lower than that of 252. These results suggested that *OsNCED1* could enhance seedling antioxidant enzyme activities under heat stress and attenuated membrane damage upon heat stress in rice seedling.

### Changes in reactive oxygen species levels in *OsNCED1* transgenic and WT seedlings under heat stress

**Figure 4:**
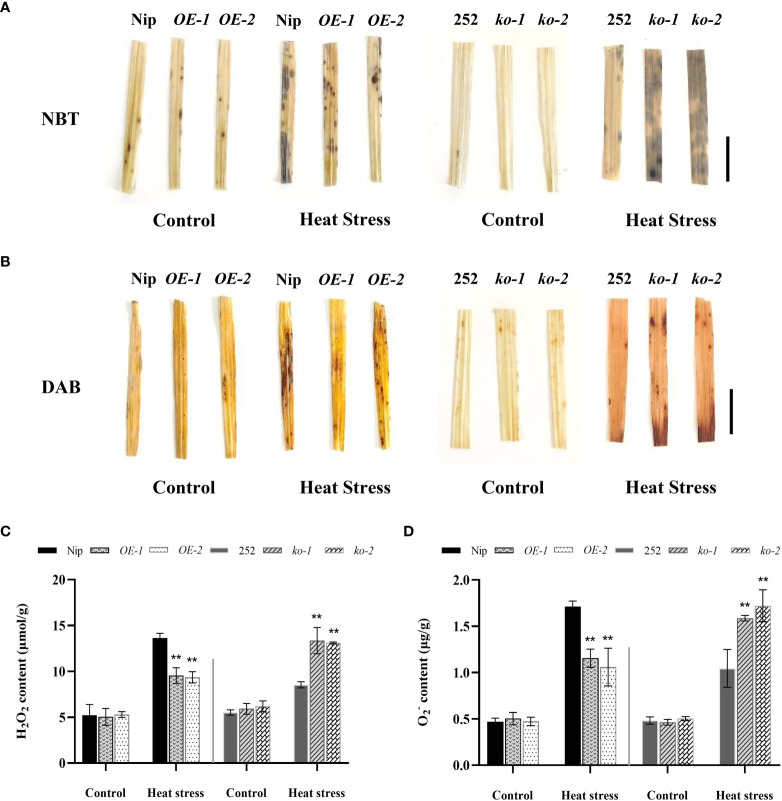
Accumulation of H_2_O_2_ and O_2_^-^ in seedlings under heat stress. **(A)** O_2_^-^ production in leaf discs of WT and transgenic lines upon heat exposure. Bar = 1 cm. **(B)** H_2_O_2_ accumulation in leaf discs of WT and transgenic lines upon heat exposure. Bar = 1 cm. **(C)** Quantitative measurement of total H_2_O_2_ content in WT and transgenic lines upon heat exposure. **(D)** Quantitative measurement of total O_2_^-^ content in WT and transgenic lines upon heat exposure. Data are means ± SD (n = 3; **P< 0.01, Student’s *t*-test).

To examine whether *OsNCED1* confers stress resistance to ROS, the accumulation of H_2_O_2_ and O_2_^-^ was examined under heat stress. The DAB and NBT staining result showed no obvious difference between the transgenic and WT seedling leaves under control conditions, whereas the leaves of the overexpression lines *OE-1* and *OE-2* seedlings under high-temperature stress stained lighter than that of Nip, whereas the leaves of the mutants *ko-1* and *ko-2* seedlings stained darker than that of 252 (**Figures 4A, B**).

Further the determination result of H_2_O_2_ content and O_2_^-^ content of *OsNCED1* transgenic and WT seedlings showed that Nip accumulated significantly more H_2_O_2_ and O_2_^-^ than that of overexpression lines *OE-1* and *OE-2*, and the H_2_O_2_ content in Nip was 43% and 46% higher than that of overexpression lines *OE-1* and *OE-2*, respectively; the O_2_^-^ content was 48% and 61% higher than that of overexpression lines *OE-1* and *OE-2*, respectively (**Figures 4C, D**). In addition, the accumulation of H_2_O_2_ and O_2_^-^ in 252 was significantly lower than that of the mutants, the H_2_O_2_ content was 57% and 54% lower than that of the mutants *ko-1* and *ko-2*, respectively; the O_2_^-^ content was 52% and 65% lower than that of the mutants *ko-1* and *ko-2*, respectively (**Figures 4C, D**). The results indicated that *OsNCED1* positively regulated plant antioxidant resistance under heat stress, and its overexpression could alleviate oxidative damage under heat stress.

### Transcriptional changes of antioxidant and defense related genes in *OsNCED1* transgenic and WT seedlings under heat stress

To elucidate the potential molecular mechanism of *OsNCED1* for heat tolerance, the transcript levels of several antioxidant and defense related genes in the transgenic and WT plants under control and heat stress conditions were detected by RT-qPCR assays. These genes included three antioxidant genes (catalase encoding gene *OsCATB*, superoxide dismutase gene *Fe^+^SOD*, and ascorbate peroxidase *OsAXP1*) (Feng et al., 2006; Ye et al., 2011; Li et al., 2015) and three defense genes (stress-responsive NAC transcription factor gene *osSNAC1*, AP2/EREBP transcription factor *OsDREB2A*, and late embryogenesis abundant enriched protein gene *OsLEA3*) (Hu et al., 2006; Xiao et al., 2007; Zhang et al., 2013), which had been shown to play critical roles in protecting rice against abiotic stress.

**Figure 5:**
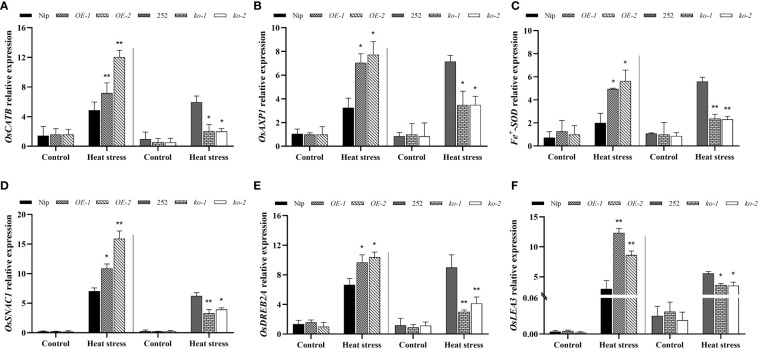
Transcriptional expression of antioxidant and defense related genes in seedlings of WT and transgenic lines under heat stress. Three antioxidant genes transcript levels of *OsCATB* **(A)**, *OsAXP1* **(B)**, and *Fe^+^SOD* **(C)**. Seedlings of WT and transgenic lines were subjected to heat stress at 45°C for 48 h, and leaves were harvested for RNA extraction, cDNA synthesis and RT-qPCR analysis. For each RT- qPCR, rice reference gene *OsActin1* was used as a control to detect its transcript levels in different samples. Data are means ± SD (n = 3; *P< 0.05, **P< 0.01, Student’s *t*-test). Three defense genes transcript levels of *OsSNAC1* **(D)**, *OsDREB2A* **(E)**, and *OsLEA3* **(F)**. Data are means ± SD (n = 3; *P< 0.05, **P< 0.01, Student’s *t*-test).

As shown in **Figures 5A–C**, there was no obvious difference in antioxidant genes between WT and transgenic lines seedlings under control conditions, but under heat stress, both WT and transgenic seedlings showed upregulated expression of antioxidant genes, especially *OsCATB*. Compared with Nip, the transcript levels of the three antioxidant related genes were significantly upregulated in the overexpression lines; the transcript levels of antioxidant related genes were significantly downregulated in the mutants compared to 252. Subsequently, the expression levels of three defense genes, *OsSNAC1, OsDREB2A* and *OsLEA3*, in the WT and *OsNCED1* transgenic lines under heat stress were examined. As shown in **Figures 5D–F**, the transcript levels of the three defense related genes were significantly higher in the overexpression lines than that of Nip under heat stress, with *OsLEA3* upregulation being the most significant. The transcript levels of defense related genes in the two mutants were significantly lower than that of 252. These data suggested that *OsNCED1* induced the expression of antioxidant and defense related genes under heat stress.

### Transcriptional changes of genes related to heat and ABA responses in *OsNCED1* transgenic and WT seedlings under heat stress

**Figure 6:**
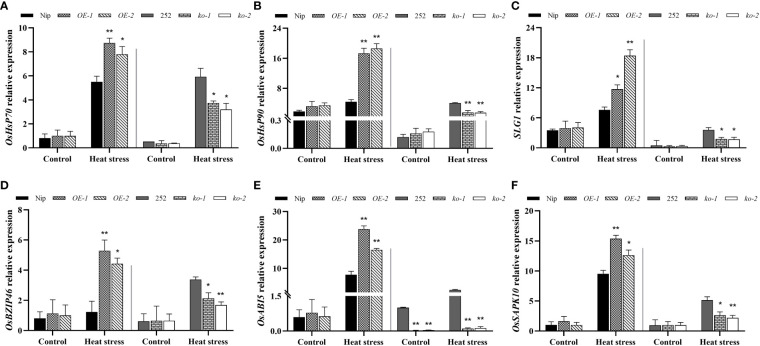
Transcriptional expression of heat tolerance and ABA related genes in WT and transgenic lines under heat stress. Three heat tolerance genes transcript levels of *OsHSP70* **(A)**, *OsHSP90* **(B)**, and *SLG1* **(C)**. Seedlings of WT and transgenic lines were subjected to heat stress at 45°C for 48 h, and leaves were harvested for RNA extraction, cDNA synthesis and RT-qPCR analysis. For each RT-qPCR, rice reference gene *OsActin1* was used as a control to detect its transcript levels in different samples. Data are means ± SD (n = 3; *p < 0.05, **p < 0.05, Student’s *t*-test). Three heat ABA related genes transcript levels of *OsbZIP46* **(D)**, *OsABI5* **(E)**, and *OsSAPK10* **(F)**. Data are means ± SD (n = 3; *p < 0.05, **p < 0.05, Student’s *t*-test).

The main function of HSPs was to regulate the folding and unfolding of proteins, as well as their subcellular localization and degradation of unfolded and denatured proteins (Singh et al., 2016). SLG1 is able to interact with cytoplasmic tRNA 2-thiolated protein 1 (RCTU1) in rice to regulate tRNA thiolation levels and thus positively regulate rice heat tolerance (Xu et al., 2020). Therefore, the expression of *HSPs* genes and *SLG1* in *OsNCED1* transgenic and WT seedlings under heat stress was examined using RT-qPCR. As shown in **Figures 6A–C**, the transcript levels of *OsHSP70*, *OsHSP90* and *SLG1* of the overexpression lines were significantly higher than that of WT under 48 h heat stress, and the transcript levels of genes related to heat tolerance in the two mutants were significantly lower than that of 252. In contrast, there were no significant difference in the expression levels of these heat tolerance related genes between WT and transgenic lines under control conditions. *OsNCED1* is involved in ABA biosynthesis, so the transcriptional change of ABA responsive genes between the transgenic lines and WT plants under heat stress was investigated. These ABA responsive genes included *OsbZIP46* (ABRE binding protein), *OsABI5* (ABRE binding factor), and *OsSAPK10* (stress-activated protein kinase), which have been shown to be involved in ABA responsive responses (Zou et al., 2008; Yang et al., 2011; Wang et al., 2020). As shown in **Figures 6D–F**, the transcript levels of the three ABA responsive genes in the overexpression lines showed greater upregulation under heat stress than that of Nip, and the mutants were upregulated less than 252. These results indicated that *OsNCED1* positively activated the expression of heat responsive genes and ABA responsive genes under heat stress.

## Discussion

Temperature is an important factor affecting rice growth and yield quality. With global warming, it is extremely important to understand how plants respond to high temperatures and breed high temperature tolerant rice varieties. Temperature of 10-15°C above ambient was generally considered as heat shock or heat stress in plants (Liu et al., 2018), whereas heat tolerance was the ability of plants to cope with heat stress, that was, the ability of plants to survive in an environment growing above the most suitable temperature (Liu et al., 2016). The optimal growth temperature at the seedling stage of rice was 25-28°C, and heat stress at seedling stage (42-45°C) lead to increased water loss, leaf wilting and yellowing, impaired root growth, and severe or even seedling death (Liu et al., 2016; Liu et al., 2018). In this study, we showed that overexpression of *OsNCED1* increased the heat tolerance of rice seedlings, while *osnced1* seedlings exhibited reduced heat tolerance (**Figure 2**), indicating that *OsNCED1* positively regulates heat stress tolerance in rice seedling plants.

ABA is a hormone that is often involved in the stress response of plants. When subjected to abiotic stresses such as cold, drought and high temperature et al., plants will rapidly accumulate ABA to activate various stress responses. For example, ABA in grapes alleviated hyperthermic damage by increasing the accumulation of osmoregulation substances and endogenous hormone content (Lv et al., 2022). And *SlSnRK2.3* regulated ABA signaling pathway regulates stomatal movement under heat stress to improve the heat tolerance in tomato (Li et al., 2022). Moreover, brassinosteroids enhanced tolerance of canola seedlings to heat stress might be due to the induction of elevated endogenous ABA concentrations (Kurepin et al., 2008). It had also been shown in rice that the application of exogenous ABA alleviated pollen sterility under high-temperature stress and was responsible for improving heat tolerance in rice by promoting sucrose transport and metabolism in spikelets, antioxidant enzyme activity, maintaining carbon balance and energy balance (Rezaul et al., 2019). And high temperature stress promoted ABA accumulation to regulate seed germination (Liu et al., 2019). *NCED* genes contributed to higher ABA levels, and increased abiotic stress tolerance in plants (Bang et al., 2013). Our results suggested that *OsNCED1* enhanced rice seedling heat tolerance, possibly by regulating the endogenous ABA content. The reasons were as follows: firstly, the transcript levels of *OsNCED1* in both WT plants were rapidly induced under heat stress (**Figure 1**). Secondly, ABA content of transgenic plants and WT increased after heat stress; while ABA content of the overexpression lines were significantly higher than that of Nip, and ABA content in *osnced1* were lower than that in 252 (**Figure 2**). Thirdly, the transcript levels of ABA related genes were significantly upregulated in the overexpression lines under heat stress (**Figures 5**, **6**). In support of this idea, previous reports had shown that ABA improved heat tolerance by inducing the accumulation of several HSPs, including HSP70 and HSP90 in plants (Li et al., 2014). There were also reports indicating that the expression of *OsLEA3* in rice seedlings was induced by ABA (Xiao et al., 2007). And Os*bZIP46* was involved in ABA signaling and abiotic stress response (Yang et al., 2011).

ROS were harmful substances reflecting oxidative metabolism. Both ROS production and scavenging affected protein, fat and nucleic acid damage and cell death (Raja et al., 2017). So, the dynamic balance of ROS was especially important for plant growth and development. Efficient enzymatic and nonenzymatic antioxidant defense systems play an important role in scavenging ROS, reducing membrane lipid peroxidation, maintaining ROS dynamic balance and redox signals (Hasanuzzaman et al., 2018). SOD dismutates superoxide to hydrogen peroxide, while peroxidases (PODs) further decompose hydrogen peroxide to water and molecular oxygen (Wang et al., 2016). MDA accumulation and the electrolyte leakage rate well reflected the degree of cell damage. In this study, the activities of SOD and POD were increased in both the transgenic and WT seedling under heat stress, and among them, the *OsNCED1* overexpression lines showed higher SOD activity and POD activity, lower MDA content, electrolyte leakage rate and O_2_^−^ content than Nip, thereby decreased cell membrane damage and oxidative stress, whereas the knockout lines happened to do the opposite, resulting in its poor survival (**Figures 3**, **4**). Consistent with the antioxidant enzyme activities, we detected that the *OsNCED1* overexpression lines showed a significant upregulation in the transcript levels of the antioxidant related genes *OsCATB*、*Fe^+^SOD* and *OsAXP1* under heat stress, and the *osnced1* mutants, although somewhat upregulated, it was not upregulated as much as 252 (**Figure 5**). Therefore, *OsNCED1* overexpression lines might be to maintain the structure and function of cells under heat stress by regulating their own enzyme system activity and content of osmotic protective substances to scavenge toxic substances such as free radicals. Consistent with this paper, it was reported that copper/zinc superoxide dismutase 1 (*CSD1*) and *CSD2* in *Arabidopsis* altered the redox status and scavenged ROS of cells to regulate heat tolerance (Guan et al., 2013). Similarly, there was also evidence that transgenic potatoes overexpressing *CuZnSOD* and *APX* had higher tolerance to high temperature and oxidative stress (Kim et al., 2010). On the contrary, the loss of *AXP1* and *CAT2* reduced the tolerance of *Arabidopsis* to high temperature stress (Vanderauwera et al., 2011). Reports had suggested that ABA in plants actively participated in antioxidant defense mechanisms through various MAPK cascades, such as ABA transiently activates MPK6 to regulate the expression of catalase 1 (CAT1), and maintaining ROS homeostasis (Raja et al., 2017).

Acting as a second messenger of ROS generating signals, H_2_O_2_ has a dual role in regulating plant physiological processes, since low concentrations of H_2_O_2_ initiate various signals in cells, whereas high concentrations of H_2_O_2_ cause oxidative damage (Quan et al., 2008; Gill and Tuteja, 2010). ABA as a stress signal plays an important role in abiotic stresses, but there are different claims about the interaction between H_2_O_2_ and ABA under abiotic stress. Our study showed that high-temperature treatment elevated endogenous ABA in the overexpression plants (**Figure 2E**), accompanied by reduced H_2_O_2_ content (**Figures 4B, C**), reduced oxidative damage, to improve heat tolerance at the seedling stage of rice. Consistent with our results, OsASR6 interacted with OsNCED1 to enhance endogenous ABA content and reduce H_2_O_2_ accumulation to improve rice salt tolerance (Zhang et al., 2022). In addition, the ABA accumulation in the *OsIAA18* overexpressing rice seedlings was significantly higher than that in WT under both salt and drought stress, and the genes of ABA synthesis and signaling pathways were also evidently upregulated, along with obviously lower level of H_2_O_2_ and improved salt and drought (Wang et al., 2021). However, it has been shown that, under drought conditions, ABA induces ROS production and increases H_2_O_2_ content in *Arabidopsis* guard cells, which activates calcium ion channels to promote stomatal closure and reduces water loss (Pei et al., 2000). It has also been shown that exogenous ABA induces H_2_O_2_ production *via OsDMI3* in rice zhonghua11 (Shi et al., 2012). We speculated that *OsNCED1* in this study might reduce membrane damage and ROS levels in plants under heat stress by regulating ABA content. However, the exact role of *OsNCED1* in ROS homeostasis under heat stress requires further investigation. Our study provides a valuable resource for the potential exploitation of *OsNCED1* in the genetic improvement of heat tolerance in rice. Future studies on *OsNCED1* will include determining how other genes, together with *OsNCED1*, are involved in other physiological functions not observed in *osnced1* mutants. In addition, other novel regulatory functions of *OsNCED1* can be investigated by identifying its interacting proteins.

## Data availability statement

The original contributions presented in the study are included in the article/**Supplementary Material**. Further inquiries can be directed to the corresponding authors.

## Author contributions

YZ and XiL: investigation, writing-original draft. WT and GZ: conceptualization, supervision. RS and XuL: formal analysis. FW, HD, YX, GC and GZ: writing-review and editing. All author contributed to the article and approved the submitted version.
