# Supplementary material for: 9-cis-epoxycarotenoid dioxygenase 1 confers heat stress tolerance in rice seedling plants
Source: Front Plant Sci. 2022 Dec 20;13:1092630. doi: 10.3389/fpls.2022.1092630 (PMC9807918; doi:10.3389/fpls.2022.1092630)
Supplement: Supplementary file 1 [file DataSheet_1.docx]

Supplementary Materials


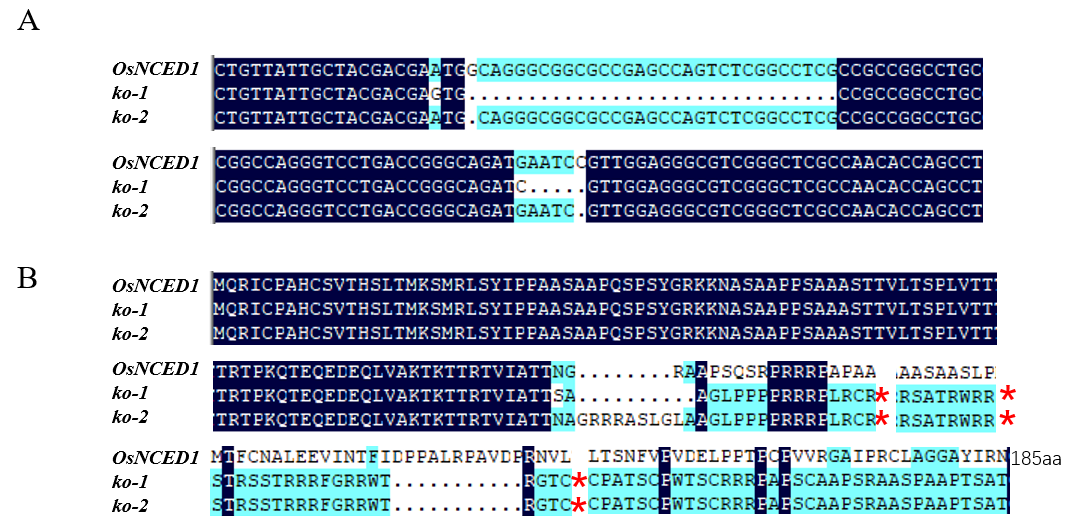


Figure S1. Knockout of *OsNCED1* in the 252 background. (A) The mutated nucleotide sequences of *ko-1* and *ko-2.* (B) The protein sequences translated by mutated nucleotide sequences of *ko-1* and *ko-2*. The red stars represent stop codons.

**Table S1**. The list of RT-qPCR primer pairs.

| Primer name | Sequence (5'–3') |
| --- | --- |
| *OsActin1-F* | GACCTTCAACACCCCTGCTA |
| *OsActin1-R* | GAGTCCAACACAATACCTGTGG |
| *OsNCED1-F* | TGACGTACTTCCGGTTCGAC |
| *OsNCED1-R* | GGAAGATGGCGTACCGCTC |
| *OsCATB-F* | GTTCGGTTCTCCACAGTCGT |
| *OsCATB-R* | CCCTCCATGTGCCTGTAGTT |
| *OsAPX1-F* | CCAAGGGTTCTGACCACCTA |
| *OsAPX1-R* | CAGTTCGGAGAGCTTGAGGT |
| *Fe^+^SOD-F* | CTTGATGCCCTGGAACCTTA |
| *Fe^+^SOD-R* | GCCAGACCCCAAAAGTGATA |
| *OsLEA3-F* | TCACTTCAAATTCGGTGCAA |
| *OsLEA3-R* | CACACCCGTCAGAAATCCTC |
| *OsDREB2A-F* | GGAATCTCCTCCTTTCATCGTG |
| *OsDREB2A-R* | TTCCGCTCCTGACAAACACG |
| *OsSNAC1-F* | CATGGTCCCGTTCTGAGGTG |
| *OsSNAC1-R* | CACACGTTGCAGCATCGATC |
| *OsHSP70-F* | GCCAAGCGTCAAGCAGTGACCAA |
| *OsHSP70-R* | GGTCATCAAAGCGCCGCCCTAT |
| *OsHSP90-F* | TTTGGGCGAAGGTGACACTGCTA |
| *OsHSP90-R* | TGGCAATGGTCCCAAGGTTCTTAAT |
| *SLG1-F* | CTACCATCGCGACTACGTCC |
| *SLG1-R* | ACTGAACGGACTTCCACACA |
| *OsbZIP46-F* | GAACACTGACTGGTCCATGCTG |
| *OsbZIP46-R* | GAGAGAAGCAACTCTGAAGCTGAG |
| *OsSAPK10-F* | TCTTGACGTGGAAAGCAGTG |
| *OsSAPK10-R* | TCCACTTCCCAGTTTCTTGG |
| *OsABI5-F* | GAAGAATTCCAGGCTACCACC |
| *OsABI5-R* | TTGATCTCAGTCCACACCTCC |
